# Supplementary material for: Disturbances of paraventricular thalamic nucleus neurons in bipolar disorder revealed by single-nucleus analysis
Source: Nat Commun. 2026 Jan 7;17:1338. doi: 10.1038/s41467-025-68094-5 (PMC12873406; doi:10.1038/s41467-025-68094-5)
Supplement: Supplementary file 1 — Supplementary Information [file 41467_2025_68094_MOESM1_ESM.pdf]

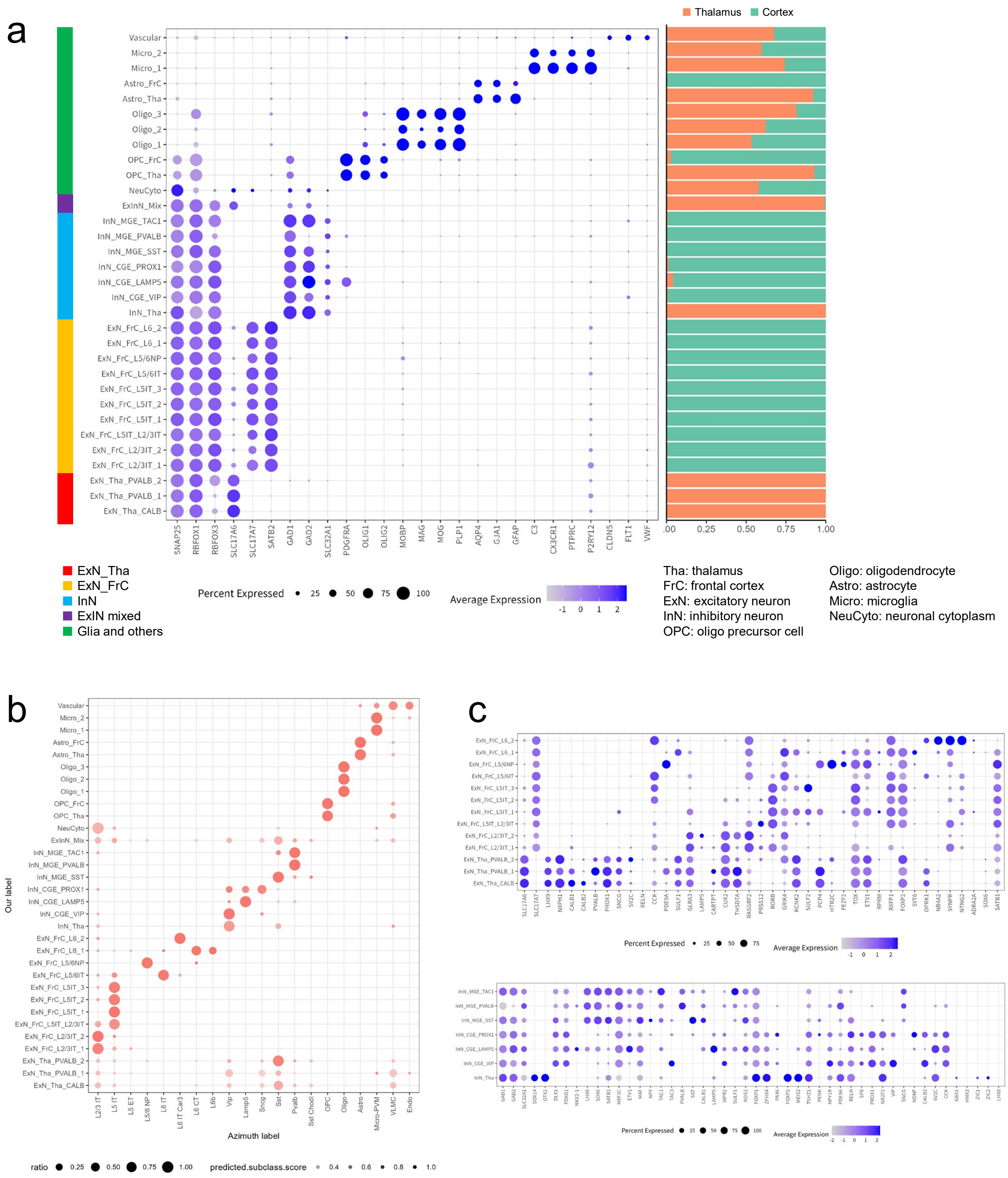

Supplementary Figure 1. Cell-type annotation of thalamic and cortical cell clusters.
a) Dot plot showing cell-type marker expression across 32 neuronal clusters in the thalamus and cortex (left), with a bar graph summarizing their relative proportions in the two regions (right). Dot size represents expression rate, and color intensity indicates average expression levels. **b)** Transfer of cell-type labels using the Azimuth pipeline. Dot size represents matching rate and color intensity indicates matching fidelity. **c)** Top: Expression of excitatory neuron (ExN) markers and selected inhibitory neuron (InN) markers within ExN clusters. Bottom: Expression of InN markers within InN clusters. NeuCyto cluster is retained as a technical reference. Source data are provided as a Source Data file.

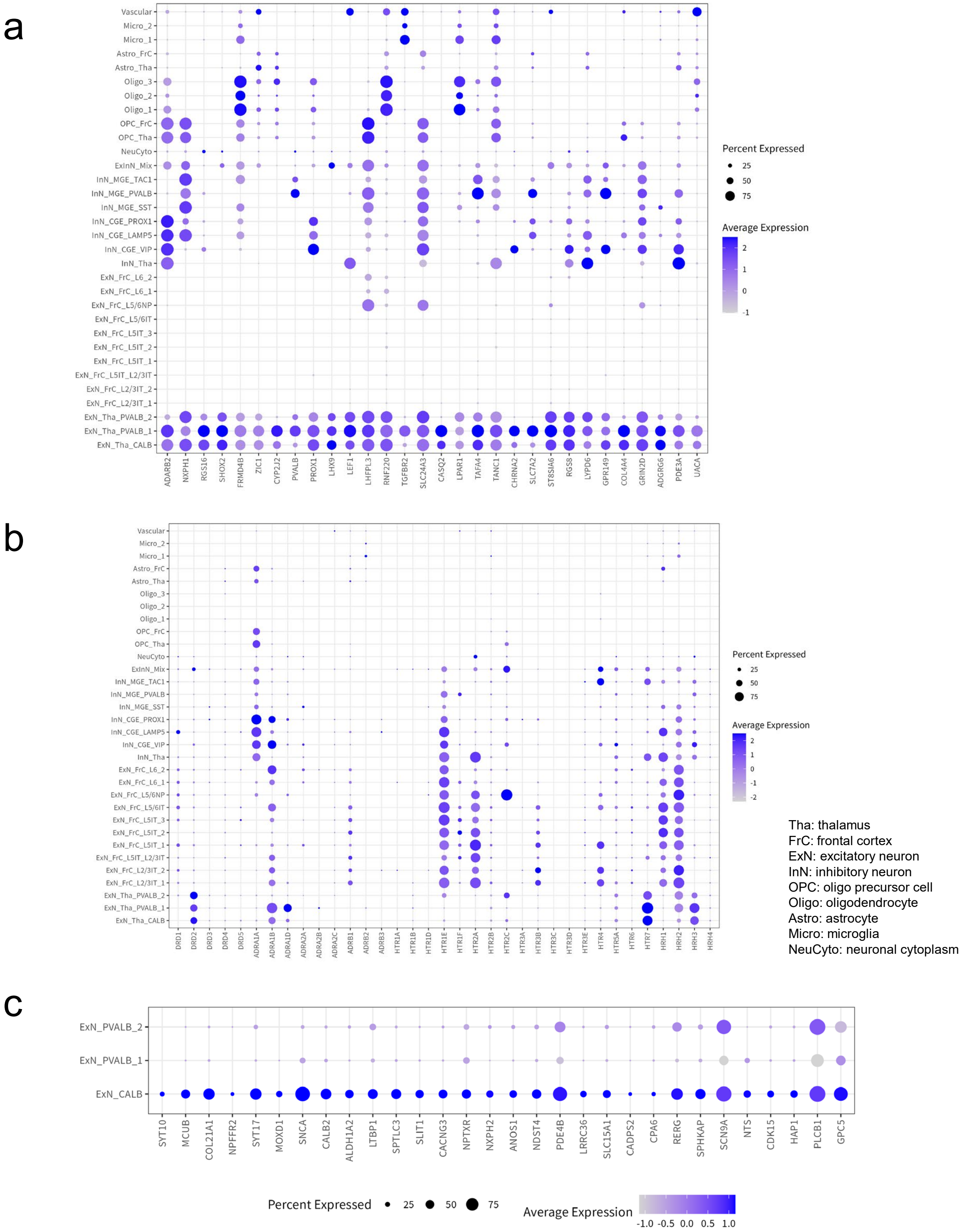

Supplementary Figure 2. Characterization of thalamic excitatory neurons and PVT neurons.
a) The top 30 ExN_Tha-specific markers distinguishing thalamic excitatory neurons from cortical excitatory neurons across the thalamus-cortex cell clusters. **b)** Expression of monoamine receptor genes. NeuCyto cluster is retained as a technical reference. **c)** The top 30 PVT-specific markers distinguishing PVT neurons from other thalamic excitatory neurons. Dot size represents expression rate, and color intensity indicates average expression levels. For a-c, dot size represents expression rate, and color intensity indicates average expression levels. Source data are provided as a Source Data file.

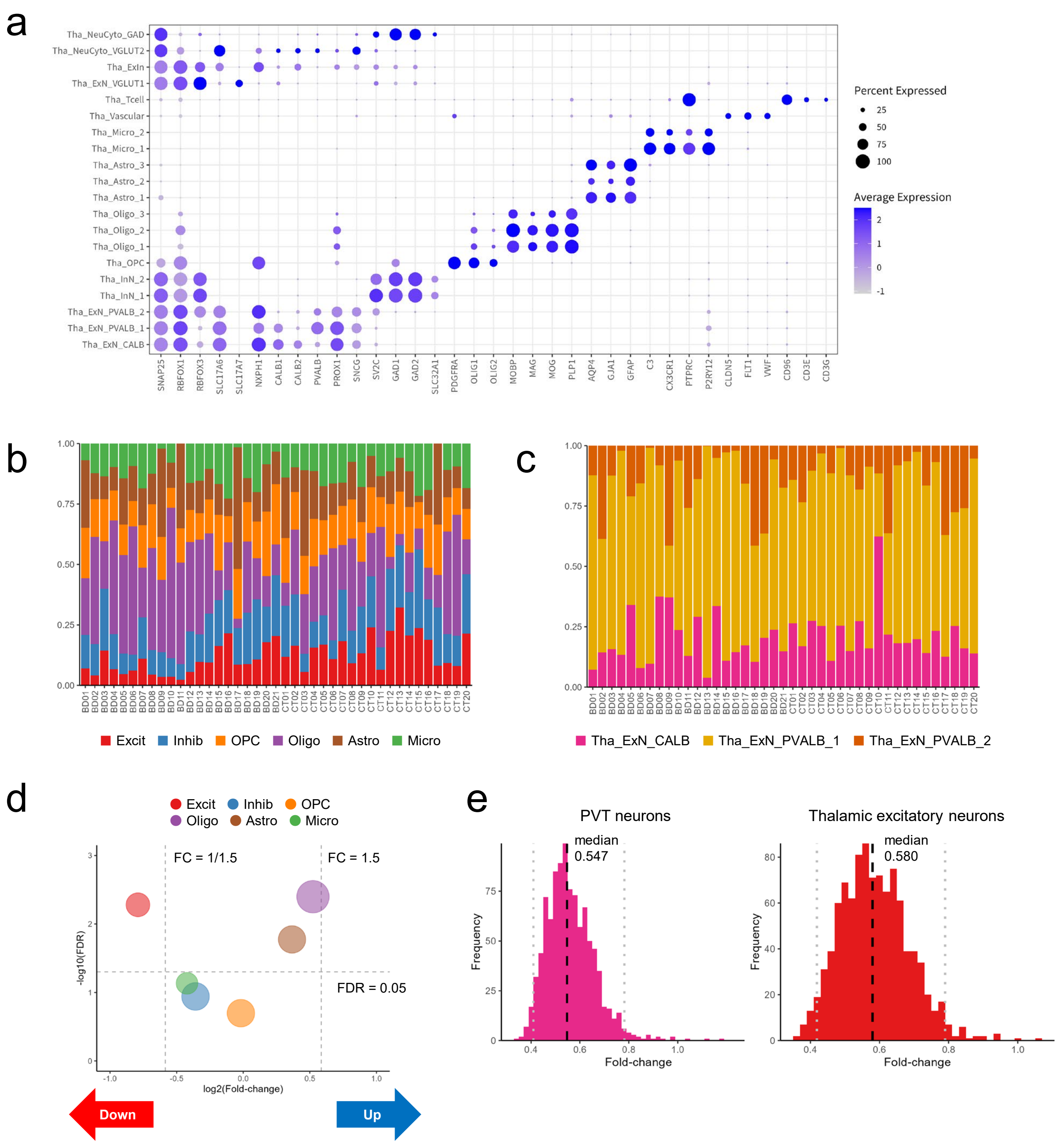

Supplementary Figure 3. Cell-type annotation in the thalamus.
a) Dot plot illustrating the expression of cell type markers across 20 clusters in the thalamus. Dot size represents the expression rate, and color intensity indicates average expression levels. NeuCyto clusters are retained as a technical reference. **b)** Proportions of major cell classes across individual thalamic samples. **c)** Proportions of excitatory neuron subtypes across individual thalamic samples. **d)** Compositional changes of major cell classes estimated by sccomp, with log2(Fold-change) on the x-axis and –log10(FDR) on the y-axis. Circle size corresponds to the nuclei count within each cluster, and color represents major cell classes. **e)** Distribution of fold-change estimates from 1,000 random sub-samplings (10 BD cases vs. 10 controls) for PVT neurons (left) and thalamic excitatory neurons (right), as determined by sccomp. Dashed bold lines indicate the median, and dotted thin lines represent the 2.5th and 97.5th percentiles. Source data are provided as a Source Data file.

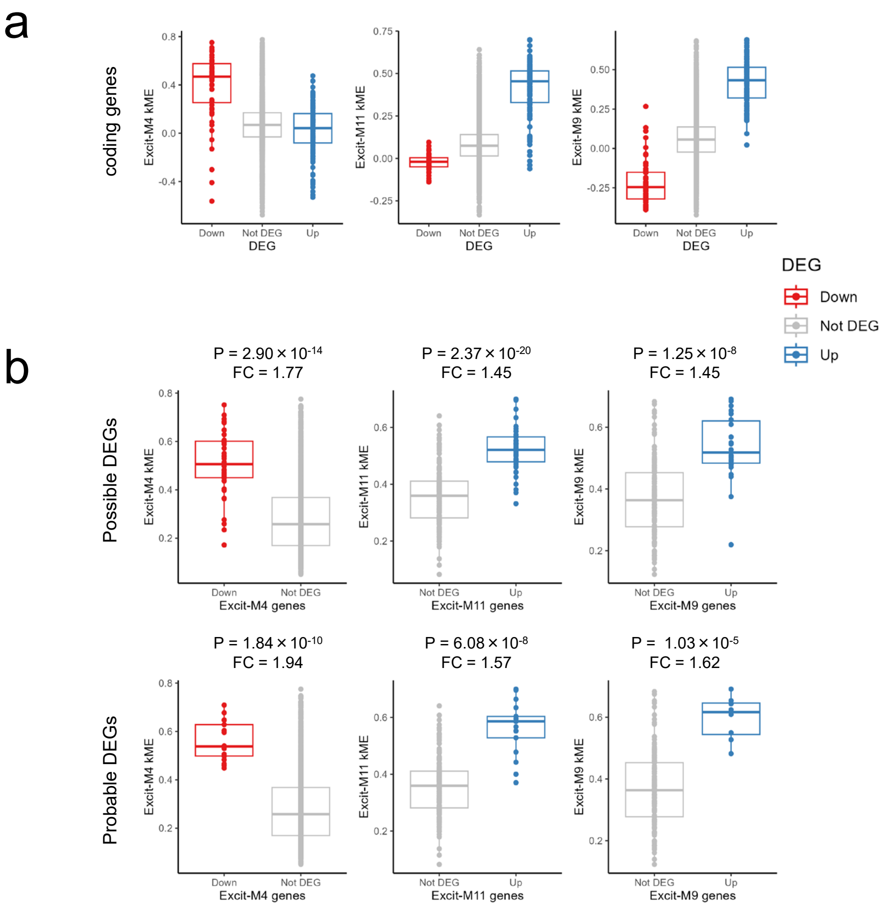

Supplementary Figure 4. Co-expression modules in PVT neurons.
a) Boxplots displaying module eigengene-based connectivity measures (kMEs) of Excit-M4, Excit-M11, and Excit-M9 for protein-coding genes in Tha_ExN_CALB (PVT neurons), excluding genes unassigned to any modules. Genes are categorized into downregulated/upregulated DEGs and non-DEGs. **b)** Boxplots showing kMEs for the genes assigned to Excit-M4, Excit-M11, or Excit-M9 in Tha_ExN_CALB. The kMEs are compared between downregulated/upregulated DEGs and non-DEGs by two-sided Welch t-tests. Box plots: The central line inside each box represents the median value, while the top and bottom of the box indicate the 75th and 25th percentiles. Source data are provided as a Source Data file.

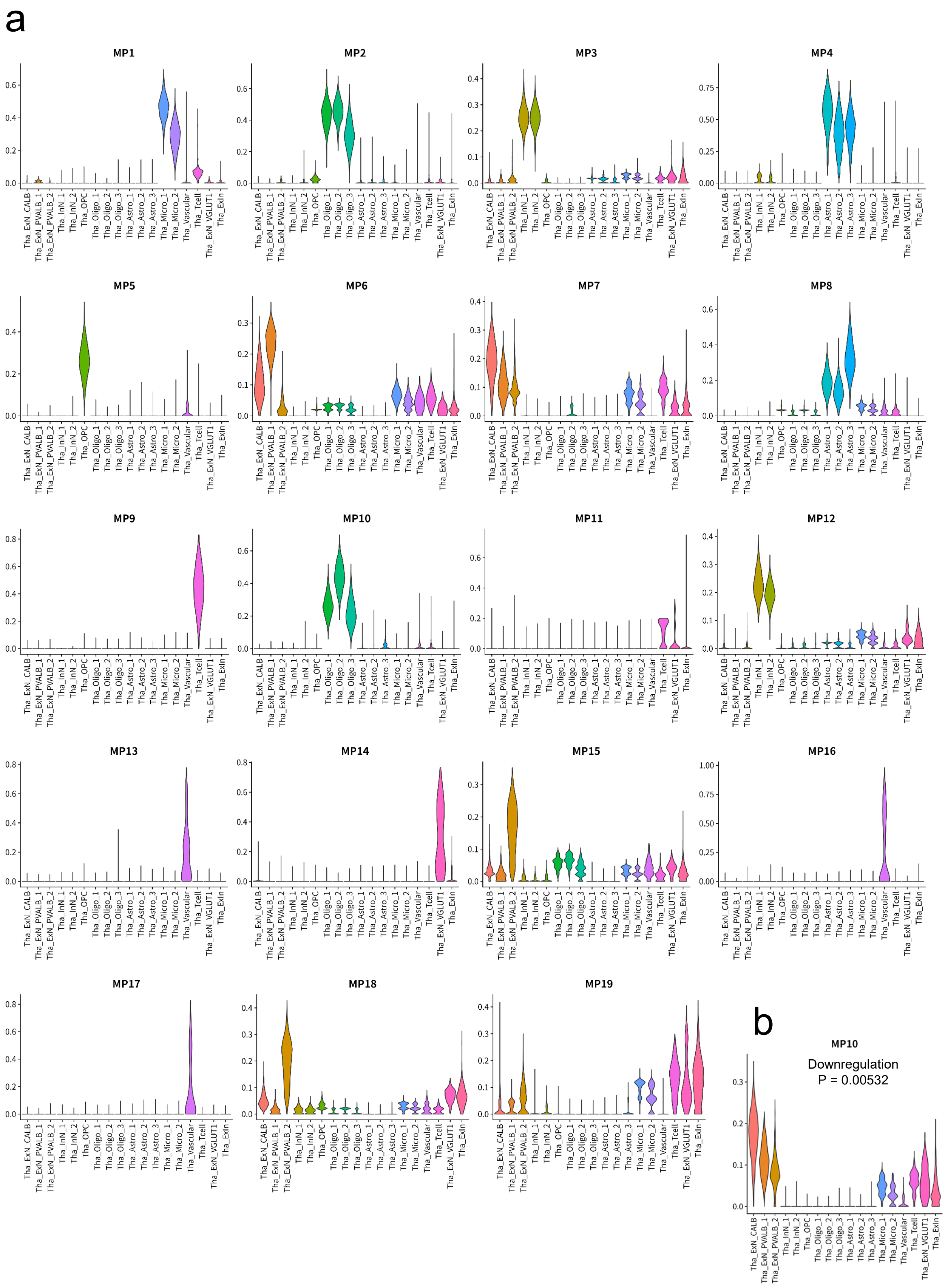

Supplementary Figure 5. Thalamic meta-gene programs estimated by geneNMF.
a) Meta-gene program (MP) scores for 19 MPs across 18 thalamic cell nuclei clusters. **b)** Trans-cellular Excit-Micro meta-gene program in another downsampled dataset (1,000 nuclei/cluster) for robustness check. P-values for downregulation in BD by GLMM are provided. Source data are provided as a Source Data file.

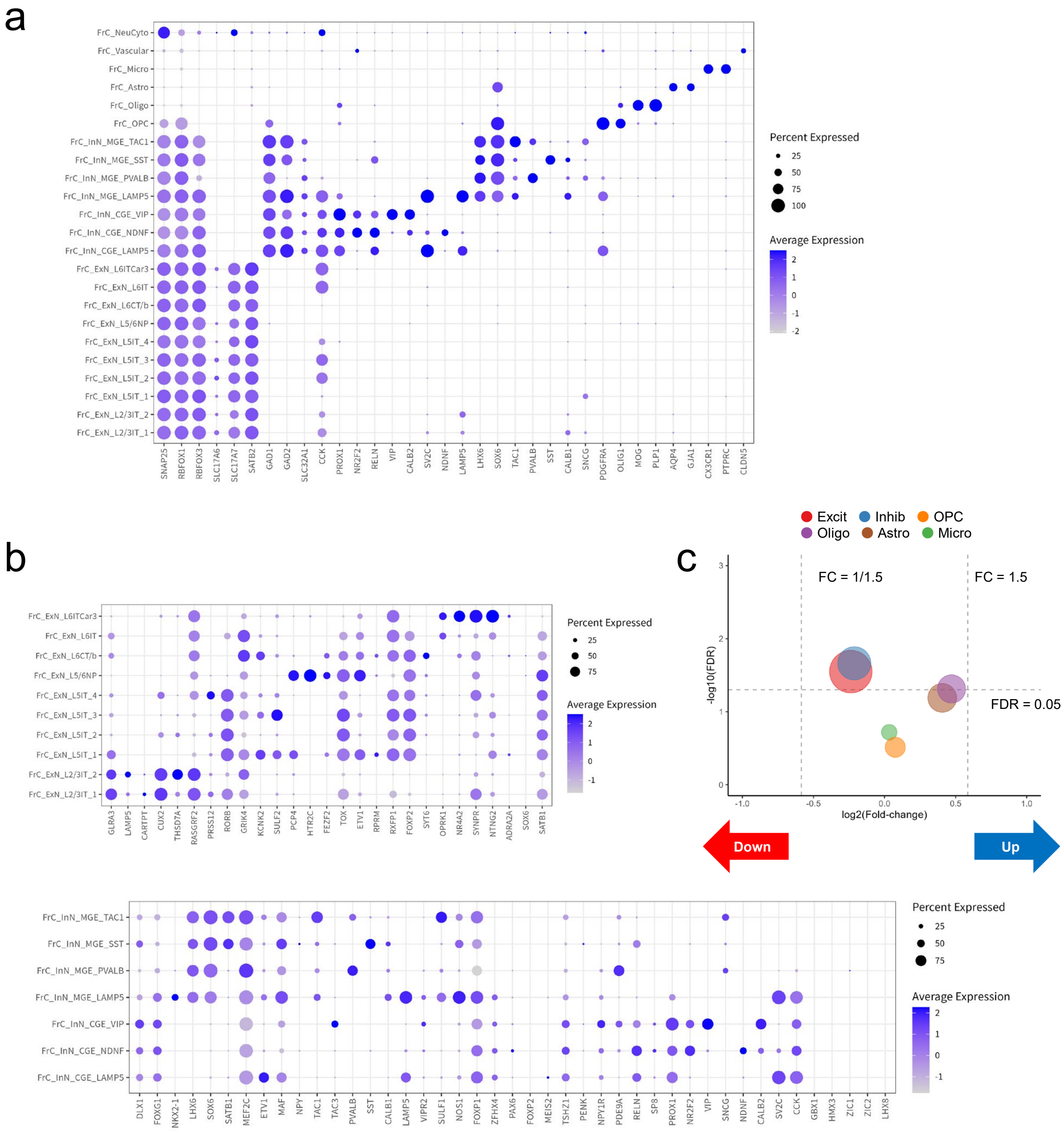

Supplementary Figure 6. Cell-type annotation in the cortex.
a) Dot plot illustrating cell-type marker expression across 23 clusters within the frontal cortex. Dot size represents the expression rate, while color denotes the average expression level. **b)** Expression of excitatory neuron (ExN) markers in ExN clusters (top) and inhibitory neuron (InN) markers in InN clusters (bottom). NeuCyto cluster is retained as a technical reference. **c)** Compositional changes of major cell classes estimated by sccomp, with log2(Fold-change) on the x-axis and –log10(FDR) on the y-axis. Circle size corresponds to the nuclei count within each cluster, and color represents major cell classes. Source data are provided as a Source Data file.

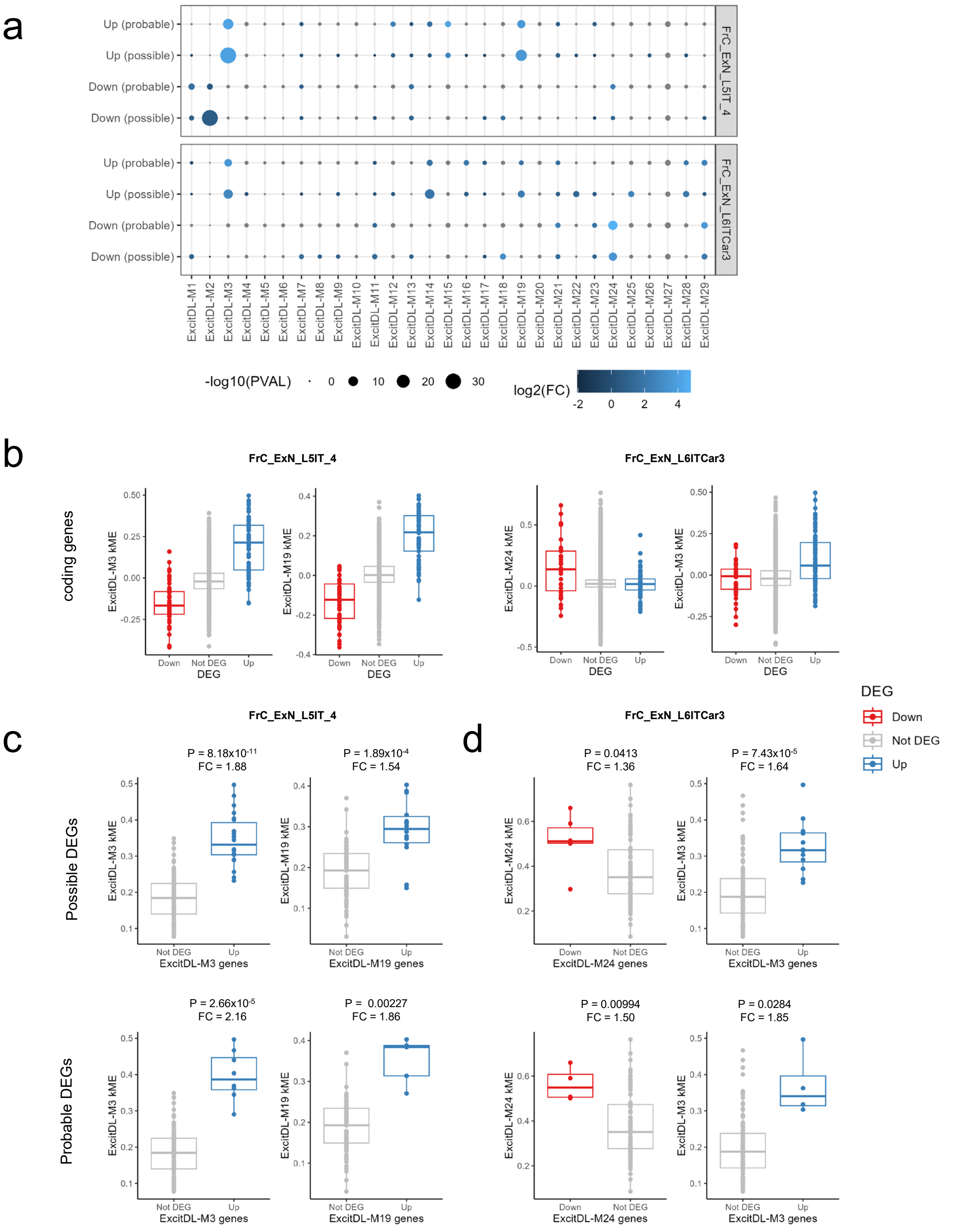

Supplementary Figure 7. Co-expression modules in cortical excitatory neurons.

a) Dot plot illustrating the enrichment of co-expression modules for DEGs in FrC_ExN_L5IT_4 and FrC_ExN_L6ITCar3. Dot size represents –log10(unadjusted two-sided P-value) from hypergeometric tests, and color denotes log2(Fold-change). **b)** Boxplots depicting module eigengene-based connectivity measures (kMEs) of enriched modules in FrC_ExN_L5IT_4 or FrC_ExN_L6ITCar3 for protein-coding genes, excluding those unassigned to any modules. Genes are categorized as downregulated/upregulated DEGs or non-DEGs. **c)** Boxplots showing kMEs for the genes assigned to Excit-M3 or Excit-M19 in FrC_ExN_L5IT_4. **d)** Boxplots showing module kMEs for the genes assigned to Excit-M3 or Excit-M24 in FrC_ExN_L6ITCar3. The scores are compared between downregulated/upregulated DEGs and non-DEGs by two-sided Welch t-tests. Box plots: The central line inside each box represents the median value, while the top and bottom of the box indicate the 75th and 25th percentiles. Source data are provided as a Source Data file.

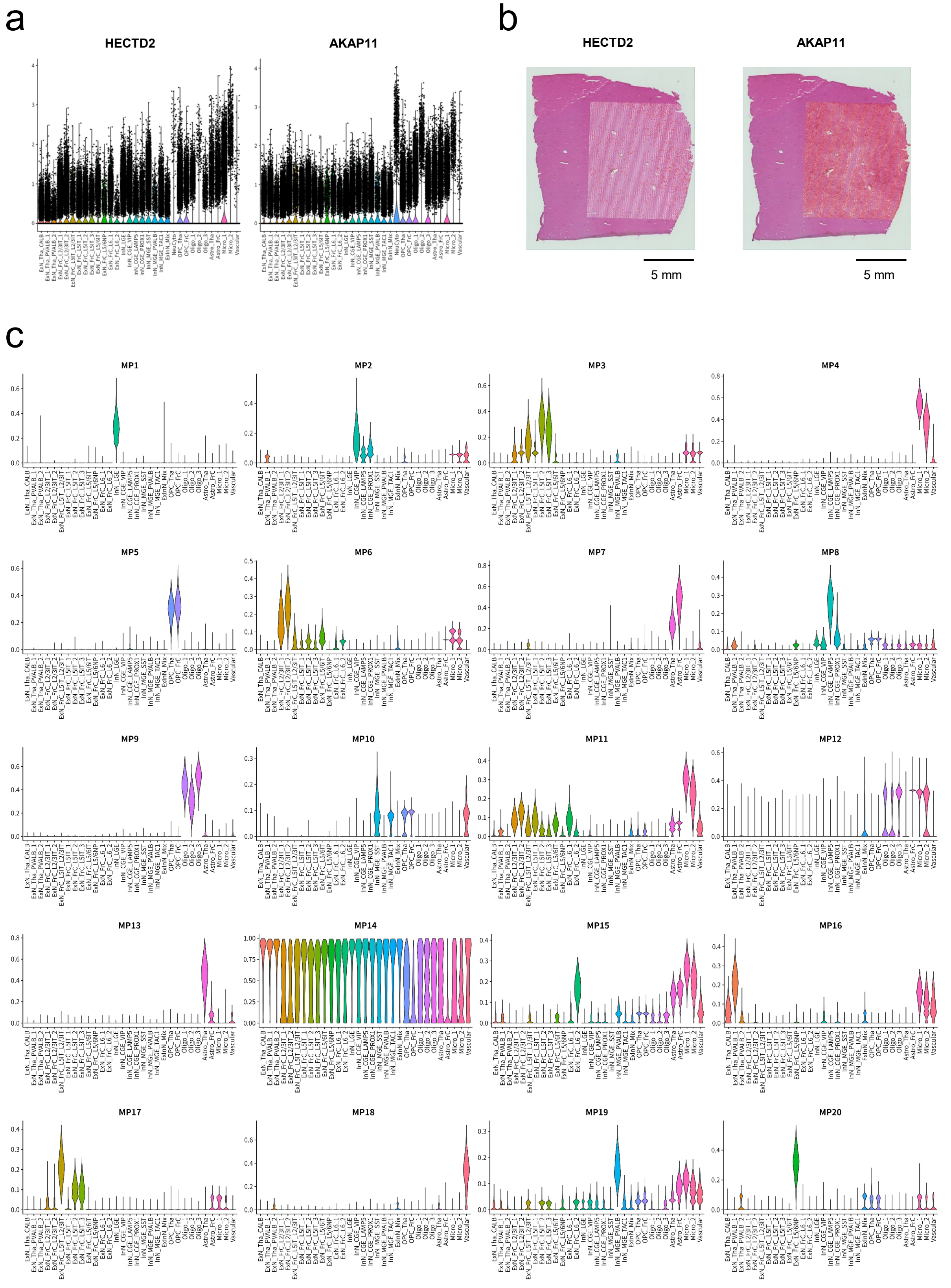

Supplementary Figure 8. *HECTD2* and *AKAP11* expression and thalamus-cortex meta-gene programs estimated by geneNMF.

a) Gene expression of *HECTD2* and *AKAP11* across the cell nuclei clusters in the thalamus-cortex integration set. **b)** Spatial gene expression of *HECTD2* and *AKAP11* in the medial thalamus. **c)** Meta-gene program (MP) scores for 20 MPs across 31 clusters in the thalamus-cortex integration set (see **Fig. 1b**). MP16 corresponds to the thalamic Excit-Micro MP, and MP11 and MP15 correspond to the cortical Excit-Micro MPs. Source data are provided as a Source Data file.

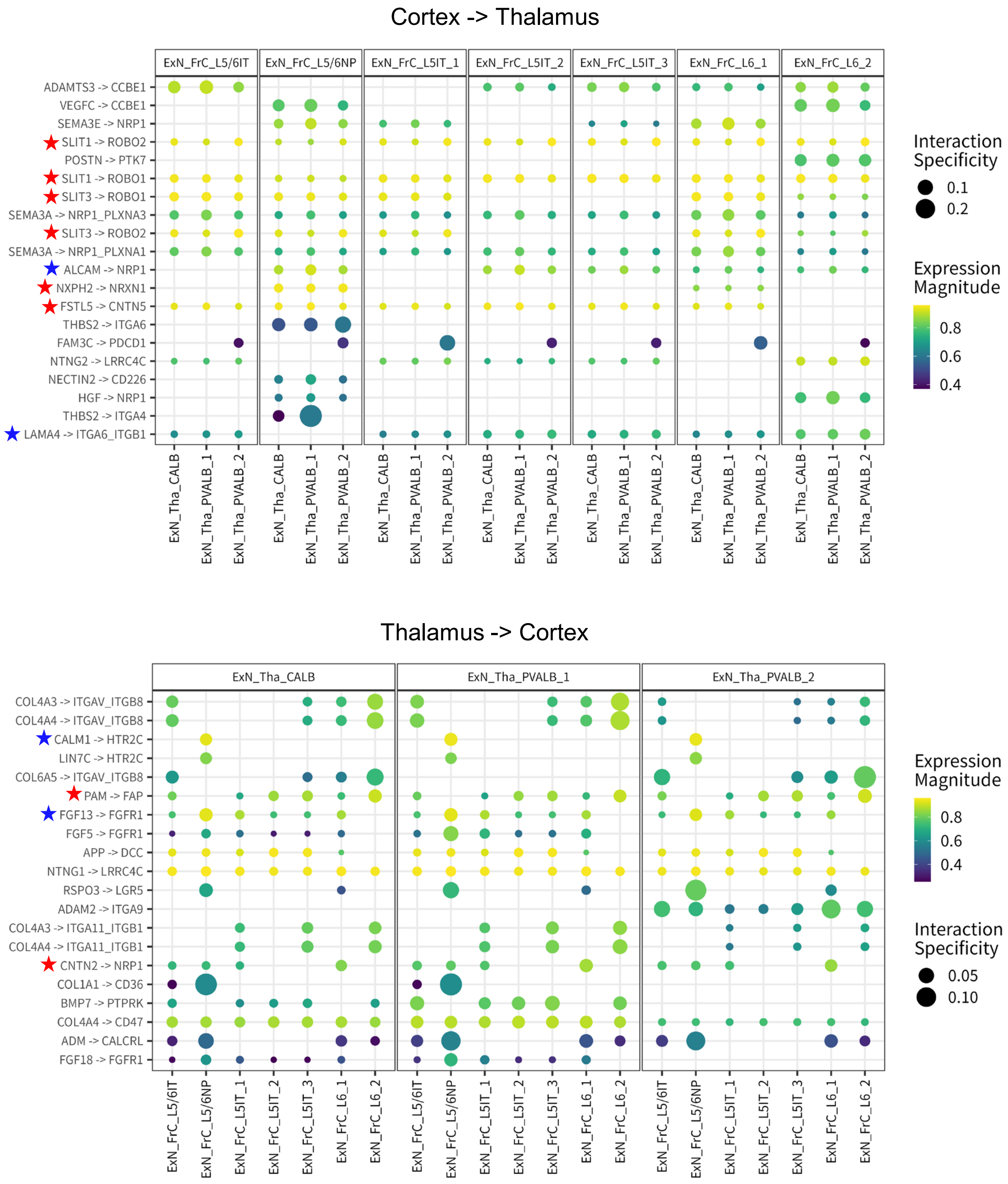

Supplementary Figure 9. Thalamus-cortex communication estimated by LIANA. Predicted ligand-receptor interactions between thalamic excitatory neurons (ExN_Tha) and cortical deep-layer excitatory neurons (ExN_FrC_DL). Dot plots represent expression magnitudes and interaction specificities, with interactions labeled as [ligand gene] -> [receptor gene]. Blue or red stars indicate ligand-receptor pairs in which the ligand or receptor is a DEG. Top: ligands expressed in ExN_FrC_DL targeting receptors in ExN_Tha. Bottom: ligands expressed in ExN_Tha targeting receptors in ExN_FrC_DL.Source data are provided as a Source Data file.

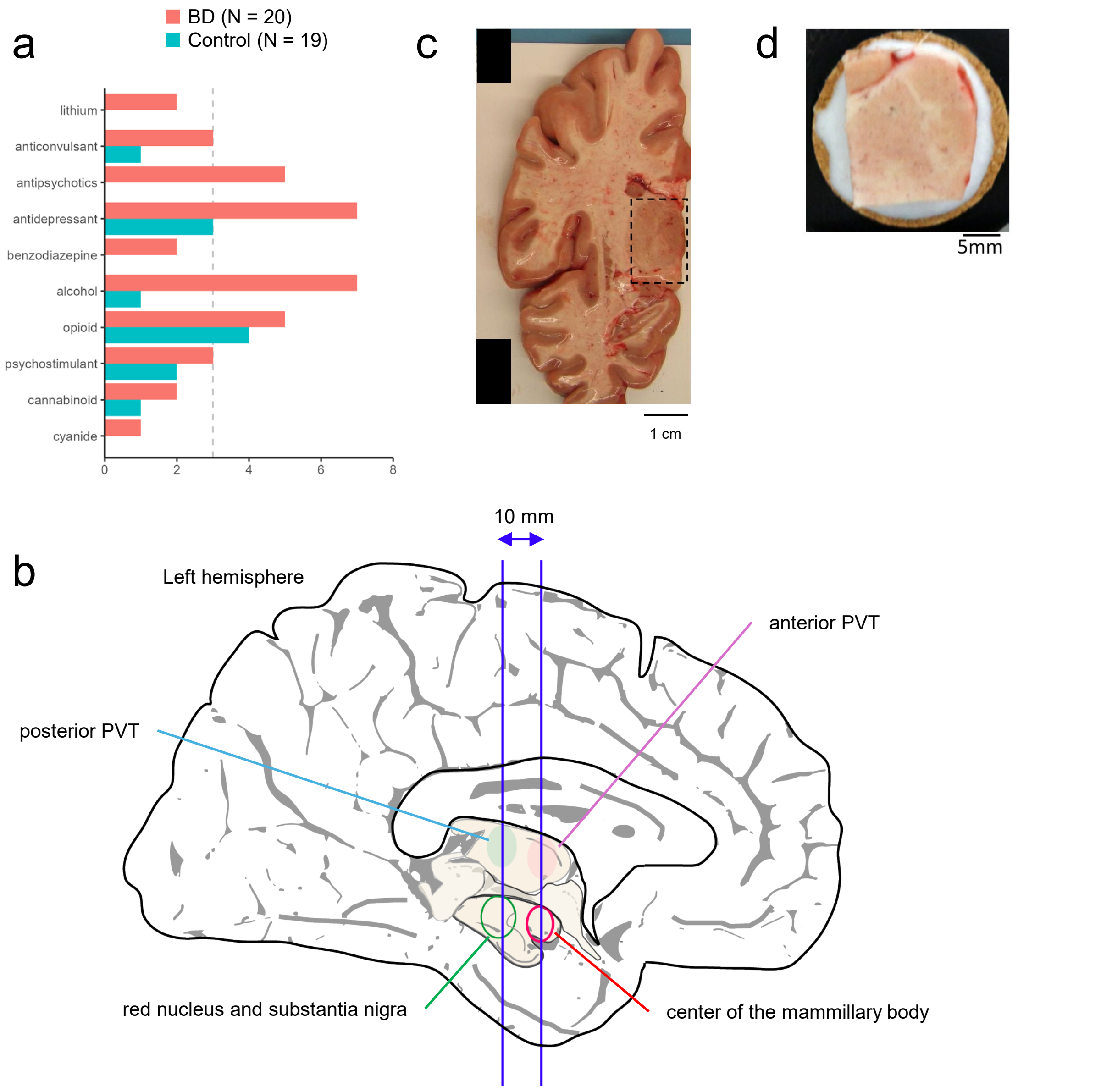

Supplementary Figure 10. Sample information and anatomical dissection.
a) Number of donors with detected substances at the time of death among 20 BD cases and 19 controls, excluding three donors with missing data. **b)** Schematic illustration of the targeted thalamic region in the left hemisphere. The 10-mm-thick slab selected for PVT dissection is indicated by violet lines. The center of the mammillary body and the red nucleus/substantia nigra, used as anatomical landmarks, are indicated by red and green circles, respectively. **c)** Representative coronal brain slab encompassing the medial thalamus (black dashed rectangle), the region of interest in this study. **d)** Representative thalamic tissue block for cryosectioning. Source data are provided as a Source Data file.

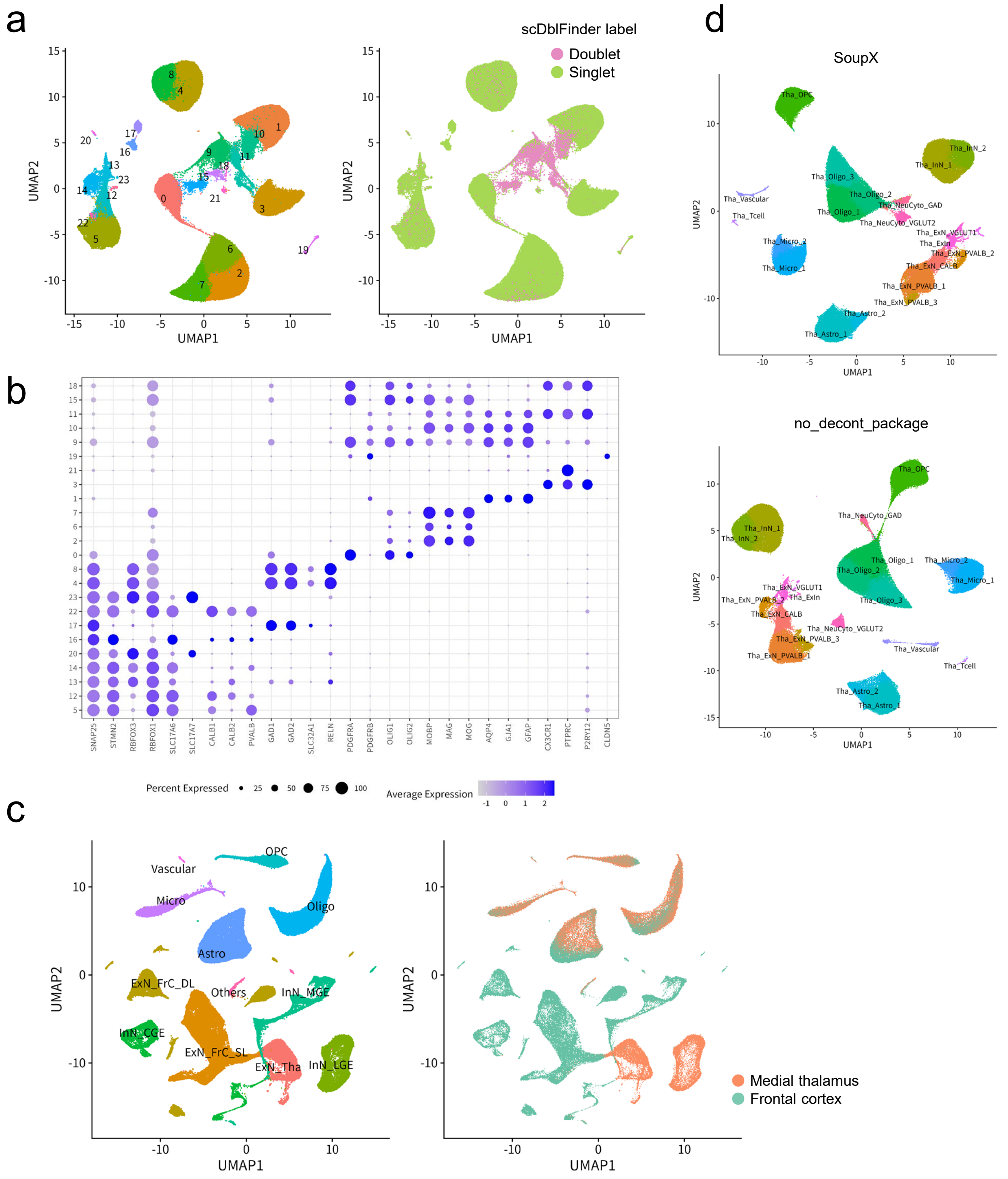

Supplementary Figure 11. Quality control (QC) for cell clustering.
a) UMAP projection illustrating thalamic cell clusters before QC (left) and their corresponding doublet/singlet status as identified by scDblFinder (right). **b)** Dot plot depicting canonical cell-type marker expression across the above clusters. Dot size represents expression rate and color intensity reflects average expression level. Clusters 9, 10, 11, 15, and 18 exhibit cell-type markers from multiple major cell classes and contain high fractions of scDblFinder-labeled doublets. **c)** UMAP projection of major cell types (left) and their respective brain region origins (right) for the thalamus-cortex integration dataset, using Seurat RPCA integration. **d)** UMAP projection displaying thalamic nuclei clusters processed with SoupX decontamination and without any ambient RNA decontamination package (no_decont_package). Source data are provided as a Source Data file.

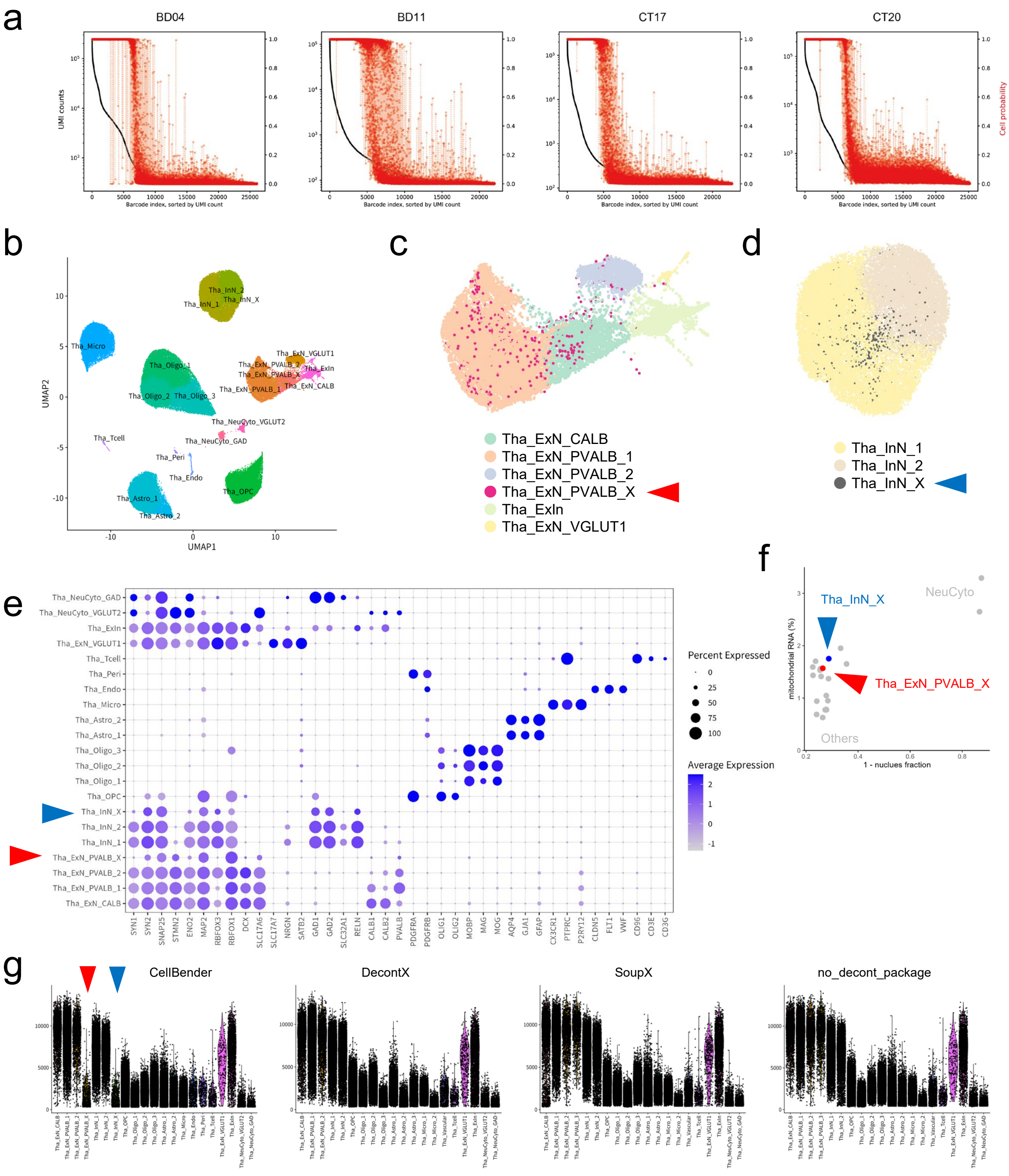

Supplementary Figure 12. CellBender-based ambient RNA decontamination.
a) Representative barcode plots illustrating unsuccessful cell probability inference by CellBender, with barcodes ranked by total UMI counts. **b)** UMAP projection of thalamic cell nuclei clustered following CellBender-based ambient RNA removal. **c)** Zoomed-in view of excitatory neuron clusters from panel b, highlighting Tha_ExN_PVALB_X using distinct coloring. **d)** Zoomed-in view of inhibitory neuron clusters from panel b, highlighting Tha_InN_X. **e)** Dot plot showing cell-type marker expression across thalamic clusters identified using CellBender. Dot size indicates the proportion of expressing nuclei, and color intensity represents average expression levels. **f)** Nuclear fractions and mitochondrial RNA fractions across CellBender-defined clusters. **g)** Number of detected genes per cluster across four RNA decontamination methods: CellBender, DecontX, SoupX, and no decontamination. Red and blue triangles indicate Tha_ExN_PVALB_X and Tha_InN_X, respectively. Source data are provided as a Source Data file.

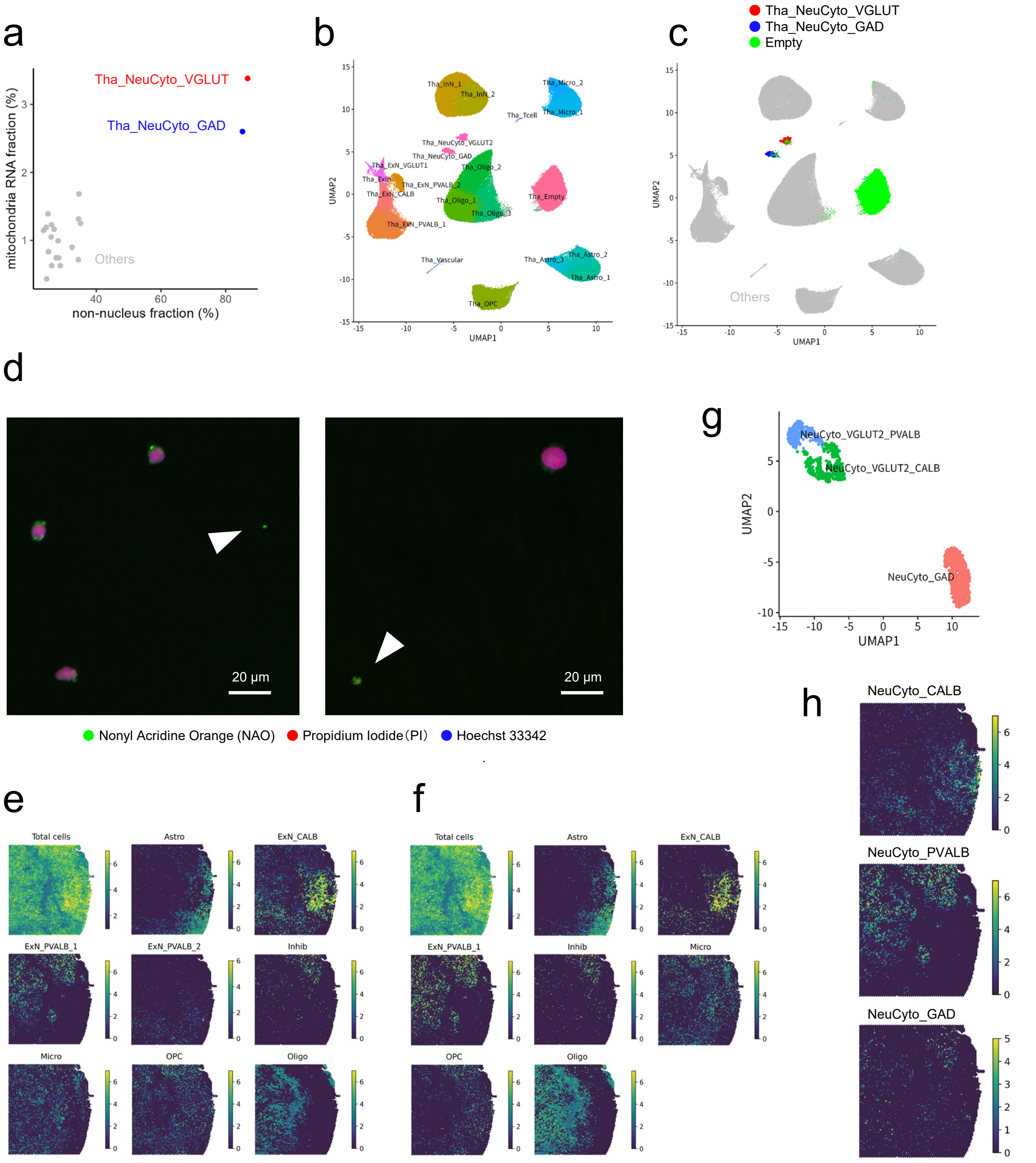

Supplementary Figure 13. NeuCyto clusters and additional spatial deconvolution.

a) Nuclear fractions and mitochondrial RNA fractions across samples in the thalamic integration dataset (see **Fig. 3a**). **b)** UMAP projection illustrating “Empty” clusters (ambient RNA, 1,000 droplets per sample) and canonical cell nuclei clusters shown in **Fig. 3a**. **c)** UMAP projection of the same clusters as in panel b, with distinct coloring to highlight Tha_NeuCyto_VGLUT, Tha_NeuCyto_GAD, and Empty clusters. **d)** Microscopic images of cytoplasmic fragments (white arrowheads) in two representative spots. Cytoplasmic fragments are indicated by NAO staining (green) in the absence of PI or Hoechst 33342 signals. Cell nuclei appear magenta due to combined PI and Hoechst 33342 staining. **e)** CytoSPACE deconvolution based solely on our snRNA-seq dataset (excluding the HBCA dataset). **f)** CytoSPACE deconvolution using only the CT06 snRNA-seq dataset. **g)** UMAP projection displaying three NeuCyto sub-clusters within the thalamus. **h)** Predicted spatial distribution of the three NeuCyto sub-clusters. Color intensity in the side bars reflects the estimated number of cells per spatial spot. For panels g and h, only control samples were used to prevent potential bias from BD-associated alterations in spatial deconvolution. Source data are provided as a Source Data file.

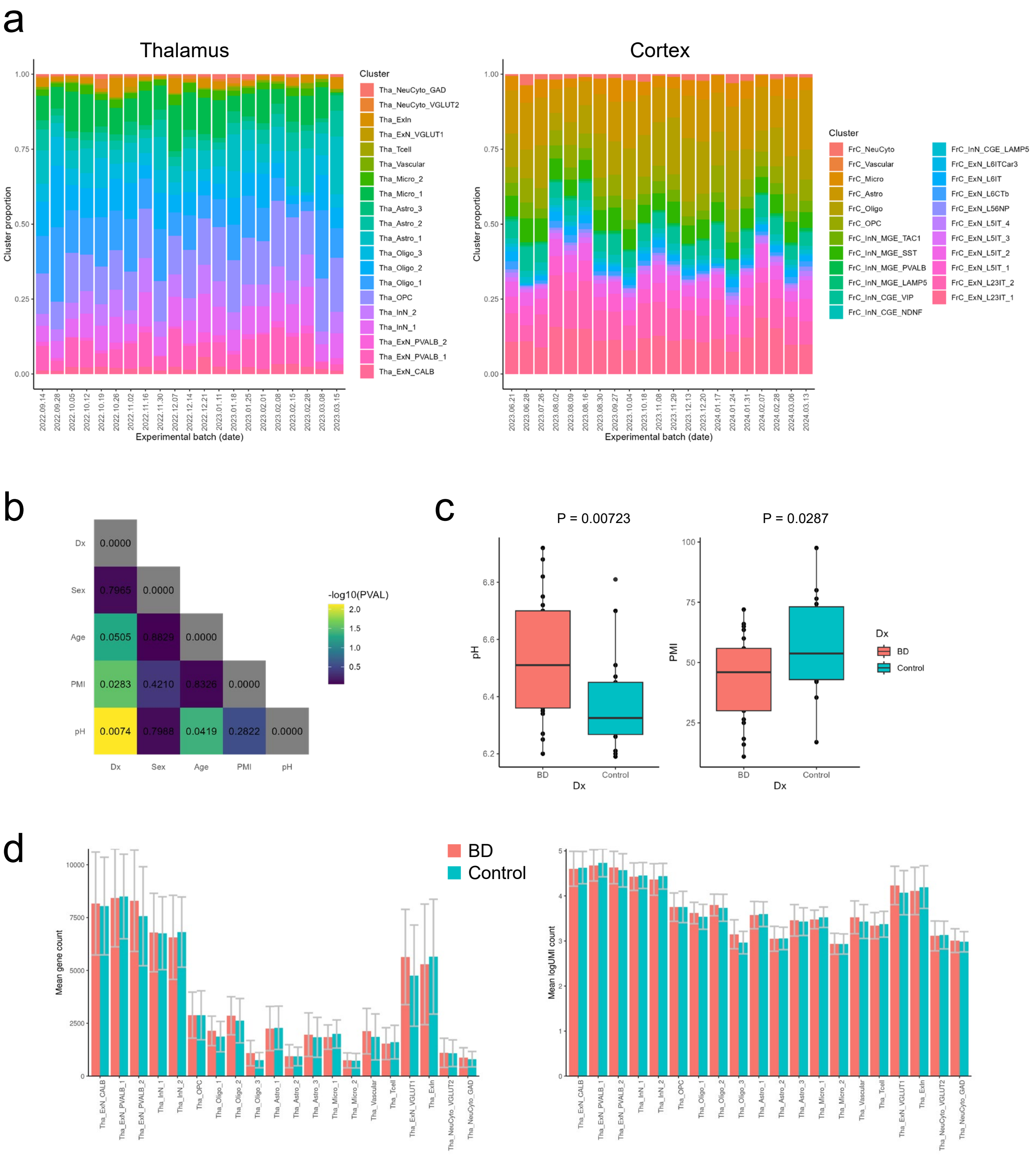

Supplementary Figure 14. Quality control (QC) for cell clustering and comparative analysis. **a)** Proportions of thalamic and cortical cell clusters across experimental batches. **b)** Heatmap illustrating the associations among potential covariates, with P-values calculated from simple regression analysis by GLM. Dx, disease status. **c)** Boxplots of pH and PMI between BD and control. The central line inside each box represents the median value, while the top and bottom of the box indicate the 75th and 25th percentiles. P-values by two-sided t-tests are shown. **d)** Mean gene count (left) and log-transformed UMI count (right) per nucleus in BD and control. Error bars indicate standard deviations. Source data are provided as a Source Data file.

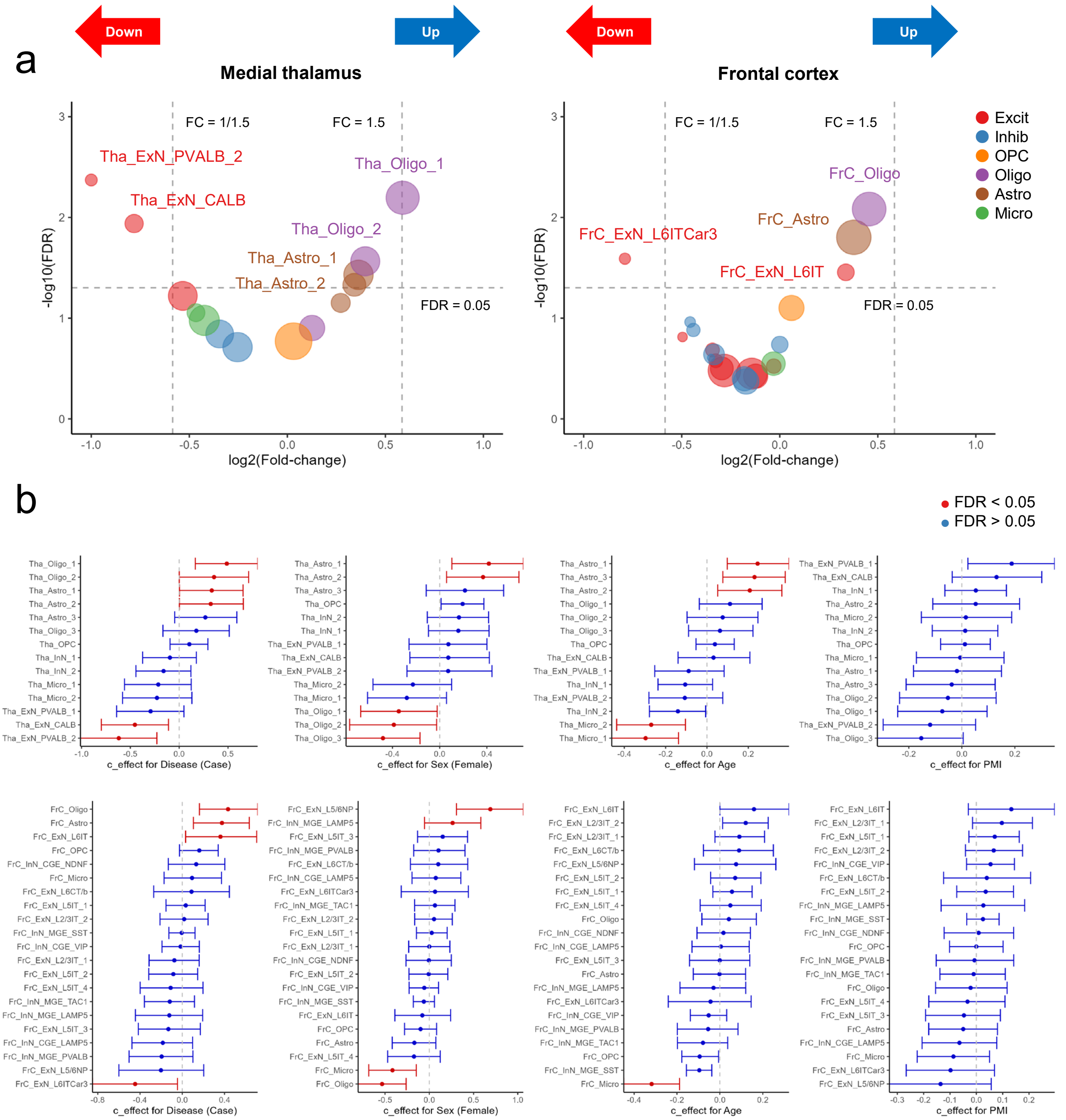

Supplementary Figure 15. sccomp analyses incorporating PMI as an additional covariate.
a) Compositional changes of cell clusters in the thalamus (left) and the cortex (right), with log2(Fold-change) on the x-axis and –log10(FDR) on the y-axis. Circle size corresponds to the nuclei count within each cluster, and color represents major cell classes. **b)** Effect (c_effect) of each covariate on cell clusters, as estimated by sccomp. The endpoints of the lines represent the lower and upper bounds of the c_effect estimate. Clusters with a false discovery rate (FDR) < 0.05 are indicated by red points and lines. Source data are provided as a Source Data file.

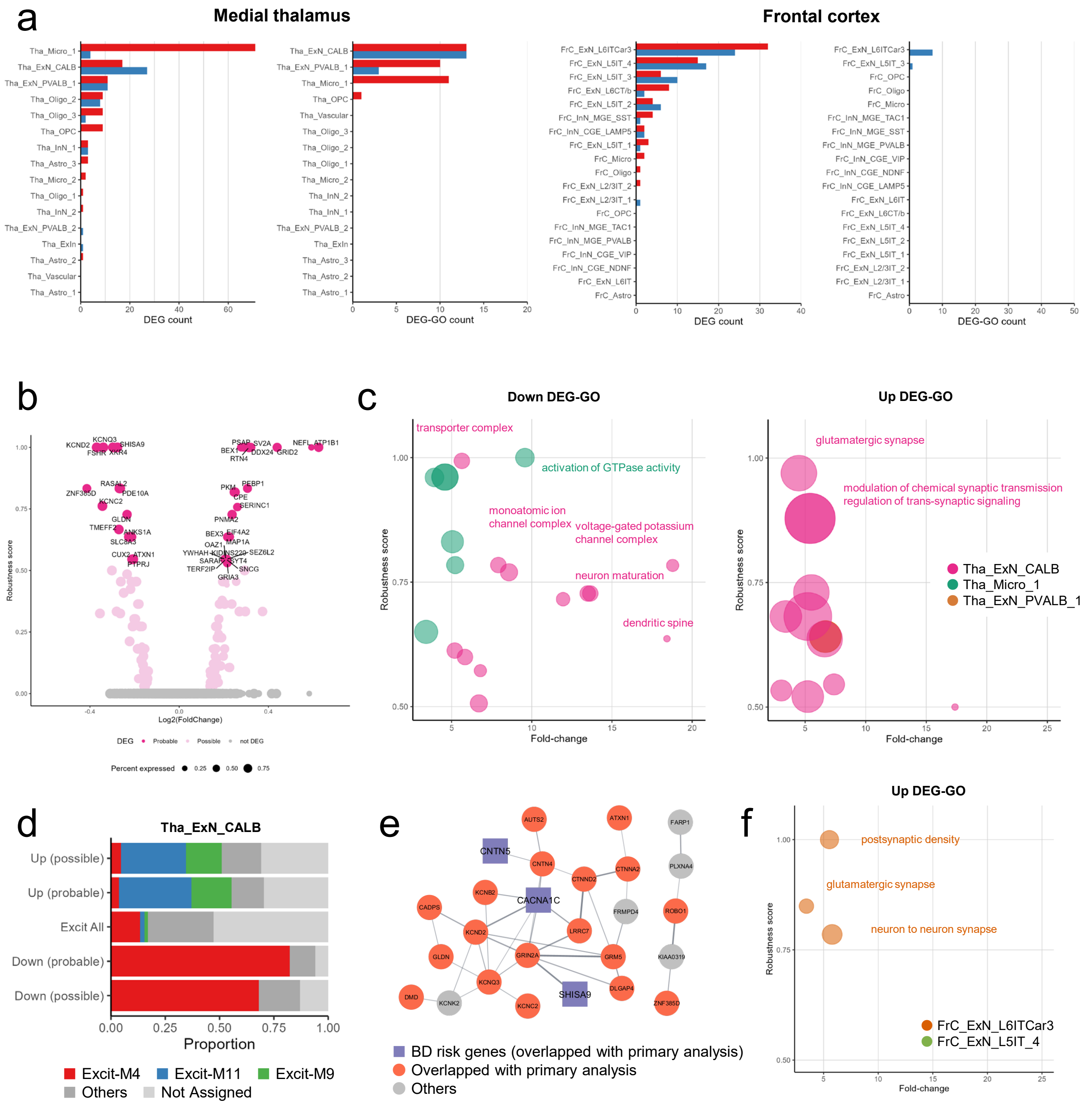

Supplementary Figure 16. DEG analyses incorporating PMI as an additional covariate.
a) DEG and DEG-GO count across thalamic and cortical clusters. **b)** Volcano plot of the genes expressed in Tha_ExN_CALB, displaying log2(Fold-change) on the x-axis and DEG robustness score on the y-axis. **c)** Downregulated (left) and upregulated (right) robust DEG-GOs in the thalamus, plotted with fold-change on the x-axis and robustness score on the y-axis. Circle size reflects the DEG count within each GO, with colors indicating respective cell clusters. Among robust DEGs, only enriched GO terms among possible DEGs are displayed for fold-change calculation. **d)** Proportions of co-expression modules in downregulated and upregulated DEGs in Tha_ExN_CALB. **e)** PPI network of downregulated possible DEGs in Tha_ExN_CALB. Colored nodes indicate overlap with DEGs from the primary analysis. **f)** Upregulated robust DEG-GOs in the cortex, plotted with fold-change on the x-axis and robustness score on the y-axis. Circle size reflects the DEG count within each GO, with colors indicating respective cell clusters. No downregulated robust DEG-GO was observed in this analysis. Source data are provided as a Source Data file.
