# Supplementary material for: Disturbances of paraventricular thalamic nucleus neurons in bipolar disorder revealed by single-nucleus analysis
Source: Nat Commun. 2026 Jan 7;17:1338. doi: 10.1038/s41467-025-68094-5 (PMC12873406; doi:10.1038/s41467-025-68094-5)
Supplement: Supplementary file 2 — Description of Additional Supplementary Files [file 41467_2025_68094_MOESM2_ESM.pdf]

Description of Additional Supplementary Files

File name: Supplementary Data 1
Description: Sample information and sequencing metrics

File name: Supplementary Data 2
Description: GO enrichment analysis by GSEA

File name: Supplementary Data 3
Description: Genes specifically expressed in ExN_Tha clusters compared with ExN_FrC clusters

File name: Supplementary Data 4
Description: Genes specifically expressed in InN_Tha clusters compared with InN_FrC clusters

File name: Supplementary Data 5
Description: Genes specifically expressed in the ExN_CALB cluster compared with ExN_PVALB clusters

File name: Supplementary Data 6
Description: Compositional changes in BD

File name: Supplementary Data 7
Description: scCODA results for thalamic and cortical clusters, including estimated fold-changes and posterior probabilities for each covariate

File name: Supplementary Data 8
Description: Immunohistochemical cell count data for thalamic excitatory neurons and oligodendrocytes

File name: Supplementary Data 9
Description: Generalized linear model (GLM) results for immunohistochemistry of thalamic excitatory neurons and oligodendrocytes

File name: Supplementary Data 10
Description: Differentially expressed genes in BD

File name: Supplementary Data 11
Description: Robust DEG-GOs in BD (FDR < 005)

File name: Supplementary Data 12
Description: Gene set enrichment analysis (GSEA) results for thalamic and cortical differentially expressed genes (DEGs)

File name: Supplementary Data 13
Description: Supplementary Table 6 Downregulated genes in Tha_ExN_CALB and Tha_Micro_1 with biological interpretability

File name: Supplementary Data 14
Description: Top 10 GSEA terms for hdWGCNA modules identified in thalamic analysis

File name: Supplementary Data 15
Description: MAGMA results for selected clusters and 10 psychiatric disorders

File name: Supplementary Data 16
Description: Overlap between our DEGs and medication signatures

File name: Supplementary Data 17
Description: Sample profiles of HBCA data used in this study, including cell cluster counts

File name: Supplementary Data 18
Description: Cell type markers used in this study

File name: Supplementary Data 19
Description: Cell cluster composition by experimental and sequencing batches in the thalamus integration set

File name: Supplementary Data 20
Description: Cell cluster composition by experimental and sequencing batches in the cortex integration set

File name: Supplementary Data 21
Description: Akaike information criterion (AIC) assessment for the GLM in compositional analysis

File name: Supplementary Data 22
Description: Analysis of variance (ANOVA) Chi-square assessment for the GLM in compositional analysis

File name: Supplementary Data 23
Description: Putative bipolar disorder risk genes identified in this study and their sources

File name: Supplementary Data 24
Description: sccomp analysis including postmortem interval (PMI) as an additional covariate

File name: Supplementary Data 25
Description: DEGs by sex, age, and PMI, using PMI as an additional covariate

File name: Supplementary Data 26
Description: DEGs by disease status, using PMI as an additional covariate

File name: Supplementary Data 27
Description: Gene ontology (GO) enrichment of DEGs by disease status, using PMI as an additional covariate

File name: Supplementary Data 28
Description: GSEA results for thalamic and cortical DEGs incorporating PMI as an additional covariate

File name: Supplementary Data 29
Description: DEGs identified using only low-PMI samples

File name: Supplementary Data 30
Description: GSEA results using only low-PMI samples

File name: Supplementary Data 31
Description: Reference list for medication signature analysis
