# Supplementary material for: Disturbances of paraventricular thalamic nucleus neurons in bipolar disorder revealed by single-nucleus analysis
Source: Nat Commun. 2026 Jan 7;17:1338. doi: 10.1038/s41467-025-68094-5 (PMC12873406; doi:10.1038/s41467-025-68094-5)
Supplement: Supplementary file 4 — Reporting Summary [file 41467_2025_68094_MOESM4_ESM.pdf]

nature portfolio

Corresponding author(s): Masaki Nishioka, Tadafumi Kato

Last updated by author(s): Dec 9, 2025

Reporting Summary

Nature Portfolio wishes to improve the reproducibility of the work that we publish. This form provides structure for consistency and transparency in reporting. For further information on Nature Portfolio policies, see our Editorial Policies and the Editorial Policy Checklist.

Statistics

For all statistical analyses, confirm that the following items are present in the figure legend, table legend, main text, or Methods section.

n/a	Confirmed	
☐	☒	The exact sample size (*n*) for each experimental group/condition, given as a discrete number and unit of measurement
☐	☒	A statement on whether measurements were taken from distinct samples or whether the same sample was measured repeatedly
☐	☒	The statistical test(s) used AND whether they are one- or two-sided<br>*Only common tests should be described solely by name; describe more complex techniques in the Methods section.*
☐	☒	A description of all covariates tested
☐	☒	A description of any assumptions or corrections, such as tests of normality and adjustment for multiple comparisons
☐	☒	A full description of the statistical parameters including central tendency (e.g. means) or other basic estimates (e.g. regression coefficient) AND variation (e.g. standard deviation) or associated estimates of uncertainty (e.g. confidence intervals)
☐	☒	For null hypothesis testing, the test statistic (e.g. *F*, *t*, *r*) with confidence intervals, effect sizes, degrees of freedom and *P* value noted<br>*Give P values as exact values whenever suitable.*
☒	☐	For Bayesian analysis, information on the choice of priors and Markov chain Monte Carlo settings
☐	☒	For hierarchical and complex designs, identification of the appropriate level for tests and full reporting of outcomes
☐	☒	Estimates of effect sizes (e.g. Cohen's *d*, Pearson's *r*), indicating how they were calculated

Our web collection on statistics for biologists contains articles on many of the points above.

Software and code

Policy information about availability of computer code

Data collection	No software was used for data collection.
Data analysis	All software used in this study is publicly available:<br>Cell Ranger (v7.0.0), DecontX in celda (v1.16.1), scDblFinder (v1.14.0), Seurat (v5.0/v5.1), Harmony (v1.2.0), clusterProfile1 (v4.10.1), SpaceRanger (v2.0.1), Loupe Browser (v8.0.0), CytoSPACE (v1.1.0), sccomp (v2.1.6), MASS (v7.3-60), MAST (v1.28.0), hdWGCNA (v.0.3.03), GeneNMF (v0.6.0), lme4 (v1.1-35), LIANA (v0.1.13), QuPath (v0.5.1). Fiji/ImageJ (v2.16.0/1.54p), glmmTNB (v1.1.12)<br><br>The analysis scripts are accessible at https://github.com/msk240/BDsnRNAseq (DOI: 10.5281/zenodo.17845882).

For manuscripts utilizing custom algorithms or software that are central to the research but not yet described in published literature, software must be made available to editors and reviewers. We strongly encourage code deposition in a community repository (e.g. GitHub). See the Nature Portfolio guidelines for submitting code & software for further information.

Data

Policy information about availability of data

All manuscripts must include a data availability statement. This statement should provide the following information, where applicable:

- Accession codes, unique identifiers, or web links for publicly available datasets
- A description of any restrictions on data availability
- For clinical datasets or third party data, please ensure that the statement adheres to our policy

The count data generated in this study have been deposited in the Gene Expression Omnibus (GEO) database under accession code GSE306819 [https://www.ncbi.nlm.nih.gov/geo/query/acc.cgi?acc=GSE306819]. The source data generated in this study are provided in the Supplementary Data files. The Extenal Data generated in this study are provided in figshare (https://figshare.com/articles/dataset/BDsnRNAseq/29876237). The HBCA data used in this study are available in the HBCA database [https://data.nemoarchive.org/biccn/]. The Penzo et al. data used in this study are available in the GEO under accession code GSE208707 [https://www.ncbi.nlm.nih.gov/geo/query/acc.cgi?acc=GSE208707]. The Shima et al. data used in this study are available in the DNA Data Bank of Japan (DDBJ) Genomic Expression Archive (GEA) database under accession code E-GEAD-626 [https://ddbj.nig.ac.jp/public/ddbj_database/gea/experiment/E-GEAD-000/E-GEAD-626/]. The GWAS summary statistics data used in this study are available in the Psychiatric Genomics Consortium (PGC) database [https://pgc.unc.edu/for-researchers/download-results/]. The PyschENCODE reference data used in this study are available in the PyschENCODE database [http://resource.psychencode.org/Datasets/Integrative/].

Research involving human participants, their data, or biological material

Policy information about studies with human participants or human data. See also policy information about sex, gender (identity/presentation), and sexual orientation and race, ethnicity and racism.

Reporting on sex and gender	Among the 42 donors of postmortem brain samples, 14 were female and 28 were male. We prioritized the availability of paraventricular regions of the thalamus and collected as many samples as possible. Our study is limited for the sex-specific analysis due to the small sample size of female donors.
Reporting on race, ethnicity, or other socially relevant groupings	Among the 42 donors of postmortem brain samples, 41 were supposed to be Caucasians and one Asian. We did not perform race/ethinicity-based analysis.
Population characteristics	The 42 donors of postmortem brain samples consisted of 21 patients with bipolar disorder (BD) and 21 control individuals. The individual backgrounds are detailed in Supplementary Table 1.
Recruitment	The donors were recruited by the Douglas-Bell Canada Brain Bank with the consent from the next of kin. The cause of death was not uniform between BD and control, probably affecting the results of this study. While 17 in 21 BD patients died from suicide, none in control died from suicide. We elaborate on this point in the Discussion.
Ethics oversight	Research Ethics Committee of the Faculty of Medicine, Juntendo University. Our study approval ID is M19-0278.

Note that full information on the approval of the study protocol must also be provided in the manuscript.

Field-specific reporting

Please select the one below that is the best fit for your research. If you are not sure, read the appropriate sections before making your selection.

☒ Life sciences ☐ Behavioural & social sciences ☐ Ecological, evolutionary & environmental sciences

For a reference copy of the document with all sections, see nature.com/documents/nr-reporting-summary-flat.pdf

Life sciences study design

All studies must disclose on these points even when the disclosure is negative.

Sample size	No formal statistical sample size calculation was performed, as the study relied on the availability of postmortem thalamic tissue suitable for snRNA-seq. All eligible samples were included. The final sample size is consistent with previously published case-control snRNA-seq studies of human postmortem brain tissue, which typically analyze similar numbers of donors to characterize cell-type-specific transcriptomic alterations.
Data exclusions	Among 42 individuals, one individual (CT21) was excluded from the entire analysis due to its low cell nuclei recovery rate of the thalamus samples (< 1,000 for the target of 5,000). Clusters designated as NeuCyto (neuronal cytoplasm) were excluded from the analysis because they were not cell nuclei. For transcriptional and compositional analysis, we excluded cell clusters with < 1,000 nuclei due to their low representation in each individual. The exclusion criteria was predefined.
Replication	Replication was not applicable because the study used unique postmortem human brain samples, which cannot be experimentally replicated. Each donor provides a biologically unique sample, and the analyses (snRNA-seq, histology, RNAscope) represent observational measurements rather than repeatable experimental manipulations. Instead of replication, reproducibility was ensured by using multiple independent donors per group, rigorous quality control, and statistical methods appropriate for human observational datasets.
Randomization	Sample allocation to diagnostic groups was determined by the clinical diagnosis of the donors and was not randomized, as this is an observational study using human postmortem tissue. Potential covariates including age, sex, and postmortem interval ere controlled in the

downstream statistical models.

Blinding	The experimenter was blind to sample status in the cell nuclei isolation and snRNA library preparation. The analytical pipeline was uniformly applied to all the snRNA-seq data without regard to sample status.

Reporting for specific materials, systems and methods

We require information from authors about some types of materials, experimental systems and methods used in many studies. Here, indicate whether each material, system or method listed is relevant to your study. If you are not sure if a list item applies to your research, read the appropriate section before selecting a response.

Materials & experimental systems

n/a	Involved in the study
☐	☒ Antibodies
☒	☐ Eukaryotic cell lines
☒	☐ Palaeontology and archaeology
☒	☐ Animals and other organisms
☒	☐ Clinical data
☒	☐ Dual use research of concern
☒	☐ Plants

Methods

n/a	Involved in the study
☒	☐ ChIP-seq
☒	☐ Flow cytometry
☒	☐ MRI-based neuroimaging

Antibodies

Antibodies used

PRIMARY ANTIBODIES

1) Anti-Calretinin (clone SP-13)
Supplier: Spring Bioscience (current supplier: Abcam ab16694)
Catalog number: SP-13 M3134 (Lot. 160728LVE)
Host/type: Rabbit monoclonal
RRID: Not registered
Application: IHC
Dilution: 1:500

2) Anti-VGLUT2
Supplier: Synaptic Systems
Catalog number: 135403 (Lot. 3-78)
Host/type: Rabbit polyclonal
RRID: AB_887883
Application: IHC
Dilution: 1:750

3) Anti-SOX10 (clone A-2)
Supplier: Santa Cruz Biotechnology
Catalog number: sc-365692 (Lot. F1621)
Host/type: Mouse monoclonal (IgG1)
RRID: Not registered
Application: IHC
Dilution: 1:250

SECONDARY ANTIBODIES

4) Peroxidase-conjugated AffiniPure Goat Anti-Rabbit IgG (H+L)
Supplier: Jackson ImmunoResearch
Catalog number: 111-035-144 (Lot. 132676)
Host/type: Goat polyclonal
RRID: AB_2307391
Application: IHC
Dilution: 1:1000

5) Peroxidase-conjugated AffiniPure Donkey Anti-Rabbit IgG (H+L)
Supplier: Jackson ImmunoResearch
Catalog number: 711-035-152 (Lot. 131943)
Host/type: Donkey polyclonal
RRID: AB_10015282
Application: IHC

Dilution: 1:1000

Validation

Validation followed Nature Research guidelines including:
- comparison to expected regional and cellular expression patterns,
- citation consistency with previously published applications,
- RRID registry confirmation,
- and omission negative control testing.
Staining patterns were consistent with external reference atlases (Human Protein Atlas).

PRIMARY ANTIBODIES

1) Anti-Calretinin (clone SP-13)

Validation: Validated by manufacturer and supported by peer-reviewed use in multiple tissues and applications including IHC, ICC-IF, and IHC-IF. Expression pattern consistent with known cytosol distribution. Representative references:
- Beltman et al., Virchows Arch. 2022; PMID: 35513609.
- Sakaguchi et al., Stem Cell Reports. 2019; PMID: 31257131.
- Kaulich et al., Nat Commun. 2025; PMID: 40858553.

2) Anti-VGLUT2

Validation: Widely cited (>140 publications), validated for IHC/ICC/WB. Expression restricted to expected glutamatergic regions. Registry-listed as RRID: AB_887883.
Representative reference:
- Mabry et al., World J Biol Psychiatry. 2020; PMID: 31062628.

3) Anti-SOX10 (clone A-2)

Validation: Supported by manufacturer and literature (>100 citations). Nuclear staining observed in oligodendroglial lineage as expected. Representative references:
- Keith et al., Clin Neuropathol. 2013; PMID: 24131748.
- Qian et al., Cell Res. 2020; PMID: 32561858.

SECONDARY ANTIBODIES

4) Peroxidase-conjugated AffiniPure Goat Anti-Rabbit IgG (H+L)
Validation: Registry-listed as RRID: AB_2307391. Manufacturer validation. Omission of primary antibody confirmed specificity.

5) Peroxidase-conjugated AffiniPure Donkey Anti-Rabbit IgG (H+L)
Validation: Registry-listed as RRID: AB_10015282. Manufacturer validated. Omission of primary antibody confirmed specificity.

Plants

Seed stocks

Not applicable for this study.

Novel plant genotypes

Not applicable for this study.

Authentication

Not applicable for this study.
